# Obstructive Fecalomas in an Infant Treated with Successful Endoscopic Disimpaction

**DOI:** 10.1155/2021/8815907

**Published:** 2021-02-02

**Authors:** Risa Kanai, Kengo Nakaya, Koji Fukumoto, Masaya Yamoto, Hiromu Miyake, Akiyoshi Nomura, Susumu Yamada, Akihiro Makino, Hideto Iwafuchi, Naoto Urushihara

**Affiliations:** ^1^Department of Pediatric Surgery, Shizuoka Children's Hospital, Address: 860, Urushiyama Aoi-ku Shizuoka-shi, Shizuoka 420-8660, Japan; ^2^Department of Pathology, Shizuoka Children's Hospital, Address: 860, Urushiyama Aoi-ku Shizuoka-shi, Shizuoka 420-8660, Japan

## Abstract

A fecaloma is a mass of accumulated feces with a consistency much harder than that of a fecal impaction. It is most frequently observed in the rectum and sigmoid area, and associated complications include colonic obstruction, ulceration, bleeding, and perforation. A one-year-old, previously healthy boy with no history of chronic constipation was admitted because of vomiting and abdominal distension. An abdominal computed tomography scan showed small and large bowel distension due to multiple obstructive fecalomas in the transverse colon. As the fecalomas could not be resolved by laxatives, enemas, or colonic lavage, endoscopic disimpaction under general anesthesia was attempted. Repeatedly shaving the fecalomas with biopsy forceps finally resulted in gradual fragmentation with subsequent passage. Gastrointestinal food allergy was later suggested as the cause because eosinophilic infiltration was found in a biopsy specimen of the colon wall. Endoscopic disimpaction is an effective treatment approach for addressing fecalomas to avoid more invasive surgical intervention.

## 1. Introduction

A fecaloma is a laminated mass of accumulated feces and has often been reported in patients suffering from chronic constipation [1–11]. The typical consistency of a fecaloma is more than that of a fecal impaction due to coprostasis [6]. Most cases in the literature have been reported in adults, whereas pediatric cases with no medical history are rare, especially for infants [7, 8, 11]. Fecalomas can trigger serious complications such as colonic obstruction, ulceration, bleeding, and perforation, sciatica, urinary retention, and deep vein thrombosis [2–4, 7–9]. Often, they can be treated by conservative methods; however, intractable cases sometimes require endoscopic disimpaction or surgical intervention. Herein, we report a case of multiple fecalomas with small and large bowel obstructions in an infant, suspected of having a gastrointestinal food allergy, which was successfully resolved by endoscopic disimpaction.

## 2. Case Presentation

**Figure 1:**
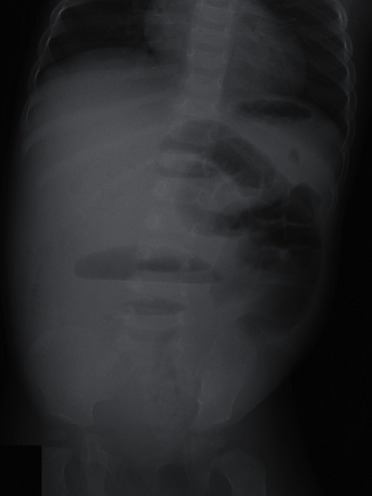
An abdominal X-ray scan demonstrated multifocal air-fluid levels.

**Figure 2:**
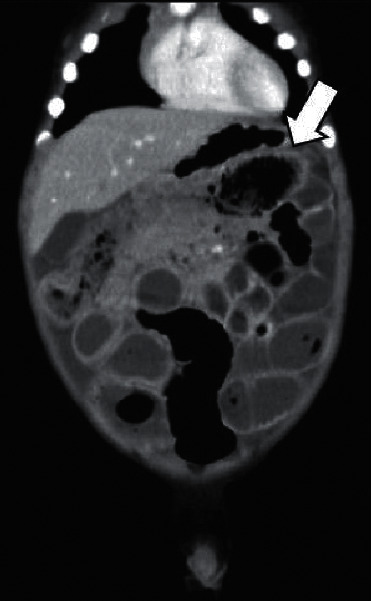
An abdominal computed tomography revealed fecalomas in the transverse colon (arrow), bowel distension from the jejunum to the transverse colon, and moderate ascites.

**Figure 3:**
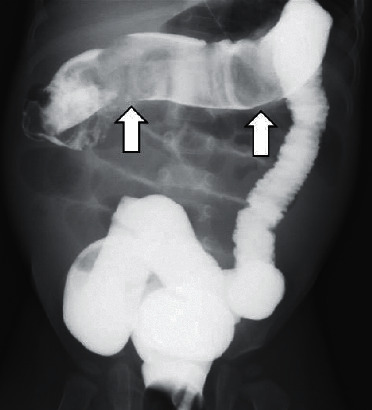
A gastrografin enema examination revealed expansion failure of the descending colon and the presence of impacted fecalomas in the transverse colon (arrows), which could not be resolved by colonic lavage.

A one-year-old, previously healthy boy with no history of chronic constipation presented with a five-day history of frequent vomiting, constipation, and abdominal distension. He was 74 cm in height and weighed 9 kg at the time of presentation. Upon examination, he was tachycardic but otherwise relatively stable. His abdomen was considerably distended but nontender and filled with palpable stool, yet a rectal exam revealed no stool. An abdominal X-ray scan demonstrated multifocal air-fluid levels (Figure 1). Suspecting mechanical obstruction, we performed an abdominal computed tomography scan. It revealed multiple fecalomas in the transverse colon, wall thickening of the descending colon, bowel distension from the jejunum to the transverse colon, moderate ascites, and no evidence of free air (Figure 2). A gastrografin enema examination revealed expansion failure of the descending colon and the presence of impacted fecalomas in the transverse colon, which could not be resolved by colonic lavage (Figure 3). His condition was not imminent, and we continued conservative observation. However, the vomiting and inability to pass the stool continued even on the following day, and his abdominal distension had worsened upon nasogastric tube drainage. He was taken to the operating room for an emergency endoscopic fecal disimpaction under general anesthesia.

**Figure 4:**
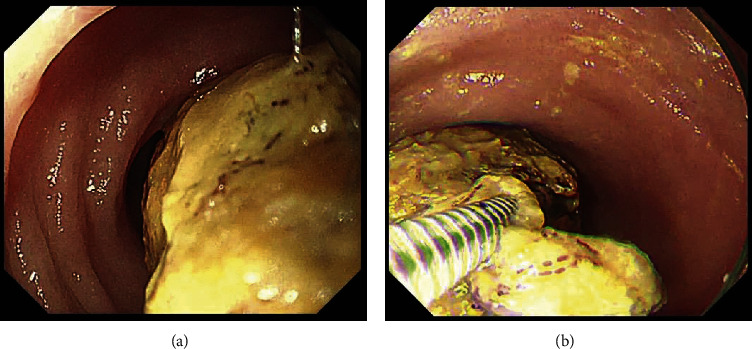
(a) A colonoscopy revealed a giant brown fecaloma occupying the lumen of the transverse colon. (b) Biopsy forceps splitted the fecaloma.

**Figure 5:**
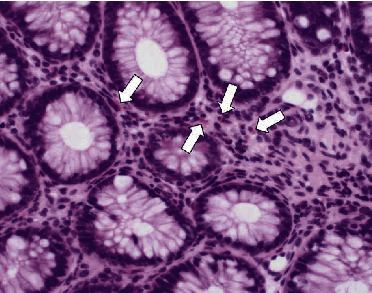
The pathology of a biopsy sample taken from the descending colon wall showed eosinophilic infiltration (arrows) (hematoxylin and eosin staining, ×400).

A colonoscopy was performed using a scope of the upper gastrointestinal tract (GIF TYPE Q260, 9.2 mm in diameter; Olympus, Tokyo, Japan) with insufflation of carbon dioxide. Edematous mucosa of the descending colon and multiple giant brown fecalomas were observed to be occupying the lumen of the transverse colon (Figure 4). Although the surface of the fecaloma was hard, large, and slippery, repeated shaving of the fecalomas with biopsy forceps resulted in gradual fragmentation. Finally, after 88 minutes, all fecalomas had been broken into fragments of a size that we thought would be able to pass through the anus. Samples were collected from the colon wall to explore the cause of the situation using biopsy. After endoscopic disimpaction, the patient experienced an intermittent bowel movement immediately, and his abdomen was less distended obviously thereafter. He was able to consume meals after recovery and was successfully dismissed from hospital care 18 days after the endoscopic treatment. Later, the pathology of a biopsy sample taken from the descending colon wall showed 22 eosinophils per high-power field (HPF), while all investigations conducted for intestinal motility disorders such as Hirschsprung's disease were negative (Figure 5). He was considered to have a food protein-induced enterocolitis syndrome, which is a kind of gastrointestinal food allergy. It was determined that, before the described event, he had eaten barley for the first time in his life. Because an allergy load test is thought to be risky in the context of severe gastrointestinal allergy, we are considering performing the test to confirm his possible barley allergy once he reaches about three years old.

## 3. Discussion

Although fecal impaction is a common and disturbing problem, a fecaloma is a particularly rare form of impaction in which a mass separable from the rest of the bowel contents is formed. Fecalomas are most frequently seen in the rectosigmoid area because the stool becomes firmer, and the colon diameter is smaller in this area [3, 9]. On the contrary, the proximal colon is a more unusual site for fecalomas, and their presence in this location can cause small bowel obstruction like seen in our present case.

Regarding the treatment of fecalomas in the rectosigmoid area, conservative procedures such as bowel rest, laxatives, enemas, manual evacuation, and colonic lavage are commonly adopted [1–3]. However, especially in the case of a fecaloma in the proximal colon or small intestine, endoscopic disimpaction or surgical intervention may be required [6, 8]. Surgical intervention sometimes becomes necessary due to difficulty with reaching these locations by endoscopy. There are some reports in the literature of fecalomas successfully dissolved by endoscopic procedures [2–5]. Recently, more creative endoscopic methods of fecaloma disimpaction using jumbo forceps or a cola injection have also been reported in [9, 10]. However, their use requires much time and effort, and one case took more than six hours to treat [9]. Therefore, endoscopic procedures should not be indicated in imminent cases.

While the majority of published cases occurred in adults, fecalomas sometimes appear in pediatric patients; however, there are no reports in which the fecalomas were dissolved by endoscopy [7, 8, 11]. We therefore believe this to be the first reported case of pediatric fecalomas successfully treated by endoscopic disimpaction. Endoscopic procedures in pediatric cases require careful maneuvering due to the fragility of these patients' intestinal tracts. In our present case, we selected the endoscope used for the upper gastrointestinal tract because of its small diameter and attempted to reduce insufflation of carbon dioxide during the procedure. Also, treatment should be performed under general anesthesia because pediatric patients are prone to poor ventilation conditions due to increased abdominal pressure; there is also a possibility of conversion laparotomy in an emergency case of perforation.

There are multiple causes of fecaloma formation, but in our case, the cause was not immediately evident as the patient had no reported history of altered bowel habits. Chronic constipation is a common problem that can lead to fecal impaction and even the development of fecalomas [1–4]. Other diseases which reported to result in fecalomas include Hirschsprung's disease, Chagas disease, psychiatric disorders, intestinal tuberculosis, and scleroderma [3, 7]. However, these causes were not apparent in our case, and a gastrointestinal food allergy was ultimately suspected because of the pathology of the biopsy specimen taken from the colon wall. Current recommendations for the diagnosis of gastrointestinal food allergy include biopsies of the intestinal mucosa that can reveal the existence of eosinophilia at a concentration greater than 20 per HPF. Our present case demonstrated 22 eosinophils per HPF and fulfilled the diagnostic criteria for a gastrointestinal food allergy. Our patient was considered specifically to have food protein-induced enterocolitis syndrome due to barley, which is a cell-mediated, nonimmunoglobulin E-mediated gastrointestinal food allergy [12, 13]. While this allergy sometimes presents together with constipation, it rarely provokes obstruction [14–17]. Some cases of gastrointestinal food allergy have required surgical intervention due to intestinal edema or obstruction [12, 13, 17]. The possible mechanism of bowel obstruction in this disease is suspected to be that inflammatory cytokines such as interferon-*γ* and tumor necrosis factor-*β* induce edema of the gastrointestinal mucosa, which causes intestinal peristalsis depression [18]. In our present case, dehydration due to persistent vomiting may also have exacerbated fecal stiffening, which rapidly accelerated the progression to fecaloma development with bowel obstruction rather than simple constipation. However, this suggestion is still speculative until an allergy load test is performed, and it also remains unclear why the patient's gastrointestinal food allergy materialized mainly in the descending colon.

In conclusion, we herein report a case of fecalomas inducing small and large bowel obstructions in an infant due to a suspected gastrointestinal food allergy. The endoscopic disimpaction of fecalomas is arduous and requires a great deal of time to perform but is an ideal treatment for use in patients without an imminent condition to avoid the need to progress to surgical intervention.

## Data Availability

The clinical data used to support the findings of this study are included within the article.

## References

[1] Wang B. T., Lee S. Y. (2019). Cecal fecaloma: a rare cause of right lower quadrant pain. *European Journal of Radiology Open*.

[2] Sakai E., Inokuchi Y., Inamori M. (2007). Rectal fecaloma: successful treatment using endoscopic removal. *Digestion*.

[3] Kim S. M., Ryu K. H., Kim Y. S. (2012). Cecal fecaloma due to intestinal tuberculosis: endoscopic treatment. *Clinical Endoscopy*.

[4] Ghosh G., Shah S., Maltz C. (2018). A case of a giant fecaloma. *Clinical Gastroenterology and Hepatology*.

[5] Sarnoff R., Girmay B., Bhakta D., Mocharla R., Williams R. (2019). An obstructing fecal bezoar in a patient with scleroderma with successful colonoscopic disimpaction. *ACG Case Report Journal*.

[6] Mushtaq M., Shah M. A., Malik A. A., Wani K. A., Thakur N. (2015). Giant fecaloma causing small bowel obstruction: case report and review of the literature. *Bulletin of Emergency and Trauma*.

[7] Parray J. S., Park T.-J., Hwa J. S., Seo J.-H., Park C.-H., Youn H.-S. (2013). Acute urinary retention in a 47-month-old girl caused by the giant fecaloma. *Pediatric Gastroenterology, Hepatology & Nutrition*.

[8] Yoo H. Y., Park H. W., Chang S.-H., Bae S. H. (2015). Ileal fecaloma presenting with small bowel obstruction. *Pediatric Gastroenterology, Hepatology & Nutrition*.

[9] Matsuo Y., Yasuda H., Nakano H. (2017). Successful endoscopic fragmentation of large hardened fecaloma using jumbo forceps. *World Journal of Gastrointestinal Endoscopy*.

[10] Kang J. H., Lim Y. J. (2014). Can fecaloma be dissolved by cola injection in a similar way to bezoars?. *Intestinal Research*.

[11] Garisto J. D., Campillo L., Edwards E., Harbour M., Ermocilla R. (2009). Giant fecaloma in a 12-year-old-boy: a case report. *Cases Journal*.

[12] Mehr S., Kakakios A., Frith K., Kemp A. S. (2009). Food protein-induced enterocolitis syndrome: 16-year experience. *Pediatrics*.

[13] Jayasooriya S., Fox A. T., Murch S. H. (2007). Do not laparotomize food protein-induced enterocolitis syndrome. *Pediatric Emergency Care*.

[14] Allen K. J., Hill D. J., Heine R. G. (2006). 4. Food allergy in childhood. *Medical Journal of Australia*.

[15] Sampson H. A. (2004). Update on food allergy☆. *Journal of Allergy and Clinical Immunology*.

[16] Nomura I., Morita H., Hosokawa S. (2011). Four distinct subtypes of non-IgE-mediated gastrointestinal food allergies in neonates and infants, distinguished by their initial symptoms. *Journal of Allergy and Clinical Immunology*.

[17] Nakaya K., Iinuma Y., Hirayama Y., Tsuruhisa S. (2016). A case of suspected gastrointestinal allergy requiring emergent laparotomy for fecal ileus. *Journal of the Japanese Society of Pediatric Surgeons*.

[18] Pohjavuori E., Viljanen M., Korpela R. (2004). Lactobacillus GG effect in increasing IFN-*γ* production in infants with cow’s milk allergy. *Journal of Allergy and Clinical Immunology*.

